# *Nocardia* spp. Pneumonia in a Solid Organ Recipient: Role of Linezolid

**DOI:** 10.1155/2018/1749691

**Published:** 2018-01-30

**Authors:** George Mwandia, Hari Polenakovik

**Affiliations:** Wright State University Boonshoft School of Medicine, Weber CHE 2nd Floor, 128 Apple St., Dayton, OH 45419, USA

## Abstract

We describe a rare infection with *Nocardia* spp. (*N. pseudobrasiliensis* species identification based on high-performance liquid chromatography analysis) in a 68-year-old renal transplant recipient. He presented with pneumonia complicated by hypoxic respiratory failure. He was allergic to sulphonamides. He was initially successfully treated with linezolid. However, he suffered severe sensory neuropathy after 4 months of therapy, necessitating linezolid cessation and completion of treatment with azithromycin. He had clinical and radiological resolution of his pneumonia and was disease free at subsequent follow-up 4 years later. This case highlights the need for alternative therapies for nocardiosis for patients that cannot be treated with sulphonamides due to allergies or/and infection with multidrug-resistant pathogens. It also illustrates the treatment limiting side effects of long-term therapy with linezolid.

## 1. Introduction

Nocardiosis is a potentially lethal opportunistic infection in transplant recipients on immunosuppressive therapy [1–4]. *Nocardia pseudobrasiliensis* is an uncommon human pathogen that is frequently multidrug resistant [5, 6]. For decades, the sulphonamide class of antibiotics has been the mainstay of therapy in nocardiosis; however, resistance to this class of drugs, allergies, or intolerances have necessitated the use of alternative antibiotics [1–4, 7, 8]. Linezolid has been shown to have promise in the treatment of nocardiosis even as monotherapy [7, 8]. Herein, we describe a case of *Nocardia pseudobrasiliensis* pneumonia treated with linezolid.

## 2. Case Presentation

The patient is a 68-year-old Caucasian male who received an allogeneic renal transplant 3 years before. He had end-stage renal disease from hypertensive nephropathy. Other comorbidities included sick sinus syndrome status post permanent pacemaker, gastroesophageal reflux disease, osteoarthritis, and depression. His medication regimen was tacrolimus, mycophenolate, prednisone, amlodipine, clonidine, metoprolol, simvastatin, aspirin, clopidogrel, calcium carbonate, magnesium oxide, and omeprazole. He is allergic to sulpha-containing drugs, furosemide and levofloxacin. He is a former smoker with no prior recreational drug use or alcohol abuse. He denies any recent travel or unusual exposures. He was admitted for a 2- to 3-week history of progressive shortness of breath, nonproductive cough, fevers, night sweats, and a 7 kg weight loss. On admission, he was pyrexial with a temperature of 39.4°C, respiratory rate of 34/min, blood pressure of 105/60 mmHg, and pulse rate of 94/min. Auscultation of the chest disclosed reduced breath sounds and crackles on the right base. A complete blood count showed neutrophilic leukocytosis of 13000/*μ*L and a total white cell count of 14100/*μ*L. Serum chemistry was significant for a high BUN-to-creatinine ratio and a hypoxic respiratory failure on arterial blood gas testing (pH 7.47, pO_2_ 40.6, and pCO_2_ 31.4 mmHg). A chest X-ray and CT chest demonstrated a right middle lobe consolidation. A CT head with contrast was negative for any cerebral mass lesion(s). The tacrolimus level was supratherapeutic at 56 ng/mL (therapeutic range 5–20). He was intubated for hypoxic respiratory failure on day 2. He underwent bronchoscopy the same day. Microbiology tests were negative for viral (adenovirus, influenza A and B, parainfluenza 1, 2, and 3, RSV, metapneumovirus, HSV, EBV, CMV, BKV), fungal (*Histoplasma*, *Cryptococcus*, and *Aspergillus* antigens), and bacterial pathogens. Bronchial washings showed an acid-fast bacillus on staining. Cultures grew variable branching filamentous bacteria after 3 days of incubation, later identified as *Nocardia pseudobrasiliensis* using high-performance liquid chromatography (HPLC method done in Quest Diagnostics Infectious Diseases, a reference laboratory in California). Susceptibility testing was done by the microbroth dilution panel using Trek RGM panel and showed multidrug resistance with susceptibility to amikacin, ciprofloxacin, clarithromycin, linezolid, and moxifloxacin (Table 1). Azithromycin susceptibility was inferred from clarithromycin susceptibility.

The patient responded to the empiric therapy with intravenous azithromycin 500 mg daily, meropenem 1000 mg 8 hourly, and linezolid 600 mg twice daily. He was extubated on day 7. Antibiotics were adjusted to oral linezolid 600 mg twice daily monotherapy on the eighth day. Tacrolimus was temporarily discontinued, and dose was adjusted to a target trough level of 5–7 ng/mL on resumption of therapy. After 4 months of linezolid treatment, the patient developed severe sensory polyneuropathy, necessitating a change to oral azithromycin 500 mg daily therapy. He completed 1 year of treatment with radiological resolution of the initial pulmonary infiltrate as early as 4 months into therapy. His neuropathy significantly improved but did not fully resolve. At 4 years of follow-up, he has no symptoms or signs of relapse.

## 3. Discussion

Among the ubiquitous saprophytic filamentous Gram-positive bacteria in the aerobic *Actinomyces* group is the species *Nocardia*, first isolated on Guadeloupe Island by Edmond Nocard in 1888 from farcy- (lymphadenitis-) afflicted cattle [9, 10]. At least 50 nocardial species have been identified as human pathogens though 113 species have been elucidated to date (see http://www.bacterio.net/nocardia.html).

*Nocardia pseudobrasiliensis* is an uncommon pathogen phenotypically similar to *N. brasiliensis* species but biochemically distinguished by its adenine metabolism and nitrate reductase activity [6, 7]. Differentiating the two pathogens is of paramount importance as *N. pseudobrasiliensis* is typically a multidrug-resistant species capable of causing invasive and disseminated diseases [6, 7]. Moreover, *N. pseudobrasiliensis* can also cause disease in immunocompetent subjects [7].

There is paucity of data on the evaluation of risk factors for nocardial infection despite the widespread practice of transplant medicine in the current era. Peleg et al. did a matched case-control analysis of 35 nocardial infections that occurred among 5126 transplant recipients over a ten-year period ending in 2005 [3]. Independent risk factors for nocardial infection in their study were the use of high-dose steroids, prior CMV infection, and high calcineurin inhibitor levels in the blood as was the case in our patient. In addition, they discovered that lung transplant recipients were the most susceptible hosts among solid organ recipients. A recent multicentre European study confirmed the findings by Peleg et al. [3]. The incubation period of nocardiosis is unknown; however, in transplant recipients, one study showed that the median time from transplantation to the development of nocardiosis was 34.1 months as was the case in our subject [1].

Due to a low prevalence of nocardiosis, there are no definitive clinical studies on the most effective antibiotics. Clinical expert opinions and conclusions from basic research or animal models are the cornerstone of recommendations, with sulphonamides being the drug of choice for at least the last six decades [1–4, 11]. Newer data show a change in the antibiotic susceptibility of *Nocardia* species. Schlaberg and coworkers analyzed susceptibility patterns of 1299 nocardial isolates comprising 39 different species, 11 of which were newly described [11]. They showed that all species were linezolid susceptible with resistance to sulphonamides 2%. The resistance to sulphonamides was even higher in *N. transvalensis* complex (19%) and *N. pseudobrasiliensis* (31%), as was the isolate in our subject [11]. Almost all species of *Nocardia* are susceptible to amikacin; however, its use in organ transplant recipients is markedly limited by nephrotoxicity [1–4].

Moylett et al. described the first successful use of linezolid for the treatment of nocardiosis in six patients, four of whom could not tolerate cotrimoxazole therapy [7]. Subsequently, several reports confirmed the efficacy of linezolid in nocardiosis [1, 8]. Linezolid is a very attractive antibiotic with excellent oral bioavailability of 100%, which allows early switch of this agent from intravenous to oral formulation [1, 7, 8]. In addition, it has extensive tissue distribution, achieving therapeutic concentrations in almost every organ in the body including the CNS [8].

However, its cost and toxicity of long-term linezolid therapy have been the main disadvantages in its widespread use [1, 8]. Substantial reduction of the cost occurred with the release of generic versions of linezolid at the end of 2015. Rare but serious neurological complications (optic and peripheral sensory neuropathies) have been reported with prolonged linezolid use and are more prevalent in subjects with preexisting neurological conditions, alcoholics, diabetics, or recipients of chemotherapeutic agents as was the case in our subject [12–15]. Duration but not the indication for linezolid use is the major determinant of neurological side effects, and studies have shown that optic neuropathies resolve after linezolid cessation, while peripheral neuropathies do not [12]. Other important side effects include myelosuppression (especially thrombocytopenia and rarely aplastic anemia) and type B lactic acidosis. [15]. These side effects appear to be mediated by direct mitochondrial protein synthesis inhibition by linezolid, and they disappear gradually after linezolid withdrawal [15].

The optimal duration of therapy is not clear, and recommendations are guided by the tendency for relapses of nocardiosis [1–4]. Pulmonary disease should be treated for 6–12 months depending on the resolution of the disease and therapeutic response, although newer studies have demonstrated favorable outcomes with shorter courses (<4 months) [2]. Our patient was treated for a longer duration than recommended due to paucity of data on azithromycin efficacy in the treatment of nocardiosis. Prolonged treatment also minimizes the risks of relapse [16]. In retrospect and based on the new European data, we believe that less than 4 months of linezolid therapy could have been adequate for our patient, and the shorter linezolid course would have prevented the evolution of sensory neuropathy.

## Figures and Tables

**Table 1:** Susceptibility testing for *Nocardia pseudobrasiliensis* (done by the microbroth dilution panel using Trek RGM panel (Quest Diagnostics Infectious Diseases)).

Antibiotic	MIC (minimum inhibitory concentration)	Interpretation
Amikacin	2	Susceptible
Amoxycillin/clavulanic acid	32/16	Resistant
Cefepime	>32	Resistant
Ceftriaxone	>64	Resistant
Ciprofloxacin	0.25	Susceptible
Doxycycline	8	Resistant
Imipenem	32	Resistant
Linezolid	2	Susceptible
Minocycline	>8	Resistant
Moxifloxacin	<0.25	Susceptible
Tigecycline	4	Resistant
Trimethoprim/sulphamethaxazole	4/76	Resistant
